# Spatial ecology, activity patterns, and habitat use by giant pythons (*Simalia amethistina*) in tropical Australia

**DOI:** 10.1038/s41598-022-09369-5

**Published:** 2022-03-28

**Authors:** Daniel Natusch, Jessica Lyons, Richard Shine

**Affiliations:** 1 School of Natural Sciences, Macquarie University, Sydney, NSW 2109 Australia; 2 EPIC Biodiversity, Frogs Hollow, NSW 2550 Australia

**Keywords:** Ecology, Zoology, Ecology

## Abstract

Although giant snakes are abundant in some tropical forests, their ecology is far less well-known than for smaller species of snakes in cooler climates. Information on spatial ecology can clarify management issues such as the sizes and types of habitats needed for conservation. We radio-tracked 27 scrub pythons (*Simalia amethistina;* snout-vent lengths 2.02 to 3.70 m) in Cape York, near the northeastern tip of Australia, for a mean period of 426 days (up to 1001 days) per snake. Home ranges were larger in males than females (means 0.60 vs. 0.28 km^2^) and overlapped considerably among individuals. All snakes used rainforest habitat, but seasonal shifts into open woodland were common. Snakes were active primarily by night, with larger snakes hunting less of the time overall, and more often by day. Hunting behaviour was seen more often during the wet season than the dry season. Average daily displacement was < 10 m, typically involving a shift from diurnal refuge to nocturnal ambush-site. A reliance on sit-and-wait predation results in small home ranges and limited movements, despite the large body size of this species.

## Introduction

All diverse biological radiations include some species that are more difficult to study than are others. Inevitably, then, our understanding of any speciose group will be based disproportionately on taxa that occur in abundance, in places where they are accessible to study^1^. For example, field-based research is more feasible if a species occurs in relatively open habitats; lives on the ground rather than above or below it; is easy to observe and capture; and occurs in sites that are close to scientific infrastructure. Such biases cannot be avoided but they mean that, wherever possible, we should also try to “fill in the gaps” by studying species that do not possess those logistically-convenient attributes. Thus, for example, we need more information on species from the tropics, because these highly biodiverse areas are less intensively-studied than are habitats in cool-temperature Eurasia and North America^2^.

Ecological research on snakes has grown rapidly over the last four decades, largely spawned by changing community attitudes and by the increasing availability of miniature radio-transmitters^3^. However, most of the species that have been studied are medium-sized terrestrial (rather than arboreal, fossorial or aquatic) colubrids, natricids, and viperids^4,5^. Most studies on giant snakes (a group restricted to tropical regions) have involved examination of field-collected specimens^6,7^ or short-term monitoring of the movements and habitat use of small numbers of individuals^8,9^. Long-term monitoring of large numbers of individuals has rarely been accomplished, because of logistical problems such as equipment malfunction and the scarcity of longterm funding for field-based research.

The scarcity of detailed ecological data on giant snakes in tropical forests poses a problem for conservation and management of these animals. Some taxa are exploited by the commercial trade in leather, meat, and/or pets, and a lack of basic information about the biology of such species has spawned disagreements about harvest sustainability^7^. The large size of these snakes suggests that they may move over large distances, an issue with strong ramifications for questions such as optimal reserve sizes^10^. Similarly, the size of area required to conserve a viable population depends upon the degree to which individuals maintain separate territories, versus overlap in space use within the same area^11^. Patterns of habitat use are important also; if a species depends upon some specific habitat type (even if that dependence is seasonal, such as for reproduction), managers need to retain sufficient areas of that habitat; or (even more challenging) ensure that a mosaic of multiple critical habitat types is maintained^12^. In the present study, we radio-tracked 27 adult scrub pythons (*Simalia amethistina*; previously *Morelia amethistina*) in a rainforest-woodland region in extreme northern Queensland, Australia, to quantify distances moved, habitats used, and seasonal and diel cycles of activity.

## Methods

### Study species

Scrub pythons are among the longest snake species in the world, with adults sometimes exceeding 5.5 m total length and 20 kg^13^. At around 700 mm total length and 50 g^14^, hatchlings of this species are larger than the adults of most other snake species^15^. Authorities disagree about the taxonomic distinctiveness of several regionally-restricted forms across northeastern Queensland and southern New Guinea, with Australian specimens identified as either *S. amethistina* or *S. kinghorni*^16^.

**Figure 1:**
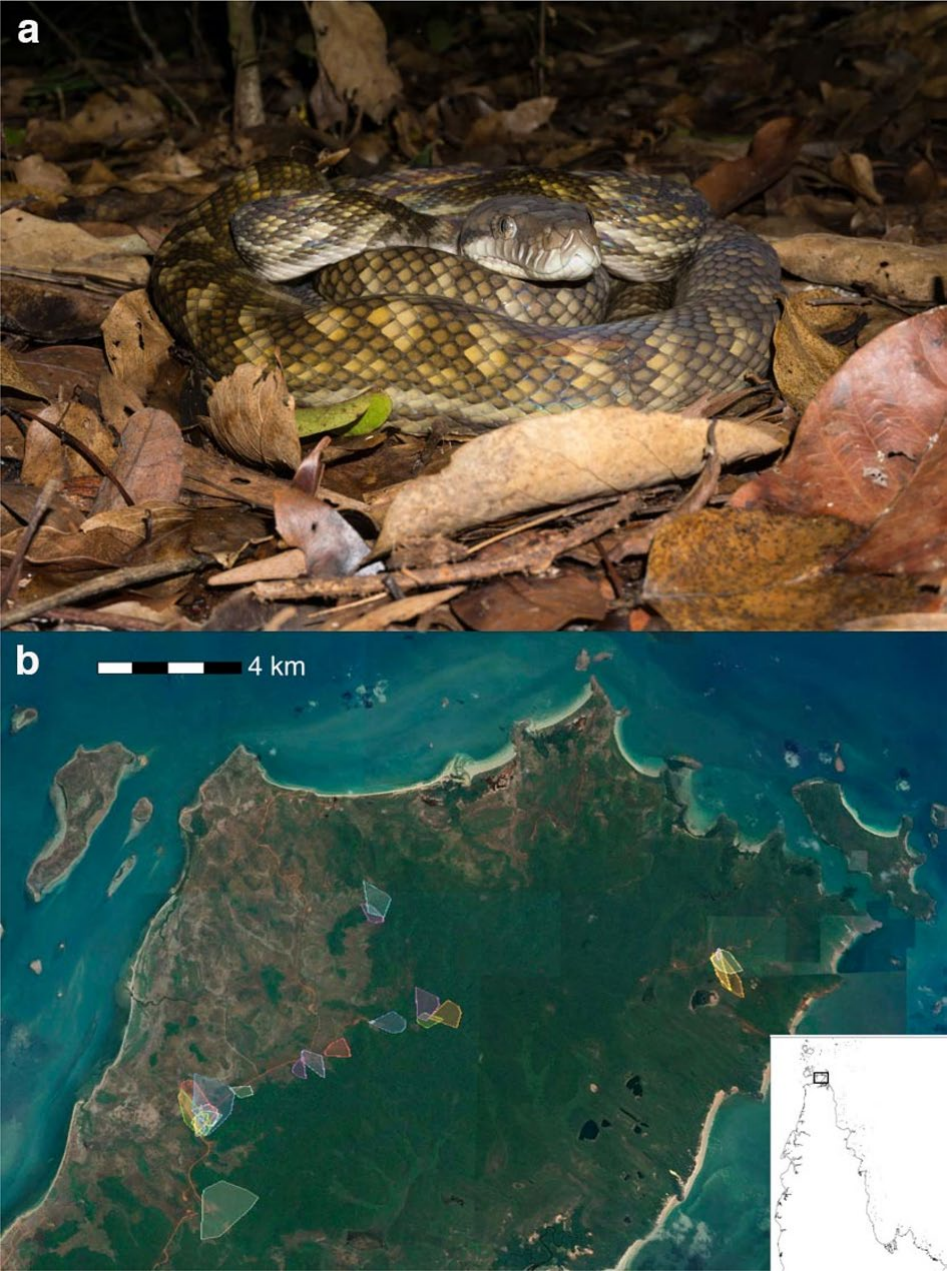
(**a**) Scrub python (*Simalia amethistina*) in hunting posture (photograph by Terri Shine); and (**b**) map of the Lockerbie Scrub in Cape York Peninsula, showing home ranges of scrub pythons (map created using the Zoatrack online platform;^29^). Dark green vegetation depicts closed rainforest habitat while brown areas depict adjacent woodlands, swamps, sand plains, and heathlands. Inset map shows the location of the study area within Cape York Peninsula, northern Queensland.

Reflecting frequent arboreality, scrub pythons are slender-bodied (Fig. 1a); a milky iridescent sheen on their scales gives them the alternative common name of amethystine python. The dorsal scales are a mosaic of brown and tan, creating a dappled colour that renders the snake inconspicuous among vegetation. Males engage in combat bouts during the mating season (in cooler months of the year) and females lay a clutch of around 12 eggs in the late dry-season, and remain with those eggs until hatching^14,17^. Scrub pythons consume a wide variety of avian and mammalian prey, and congregate under trees containing colonies of metallic starlings, feeding on nestlings that fall from the trees^18^. These pythons sometimes consume relatively large prey, including macropodid marsupials^19,20^.

### Study site

The Lockerbie Scrub is a 130 km^2^ area of semi-deciduous notophyll vine forest interspersed by tropical woodlands at the northern tip of Cape York Peninsula, Australia (10.78 S, 142.50 E)^21^ (Fig. 1b). The area is hot year-round (mean monthly minimum and maximum air temperatures from 23 to 32 °C^22^) but most rain falls during the wet season from December to April (mean rainfall 1543 mm). The remainder of the year is relatively dry (mean of 202 mm^22^).

### Protocol for radiotelemetry

We hand-captured scrub pythons from throughout the study area during nocturnal surveys of snake habitat and returned them to the laboratory for measurement and transmitter implantation. We anesthetized the pythons using vaporized isoflurane and recorded snout-vent length (SVL) using a steel ruler, body mass using PESOLA® spring scales, and sex by probing the cloacal bursae and recording depth. Each snake was then fitted with a radio-transmitter (AI-2, Holohil Systems Limited, Ontario, Canada) that was surgically implanted into the body cavity following the procedure described by Whitaker & Shine^23^. Transmitters weighed between 17 and 28 g (with smaller snakes receiving smaller transmitters) and ranged from 0.19 to 1.9% of snake body mass. This is a small burden compared to the size of meals often consumed by this species (prey items sometimes weigh more than the predator^20^). We released all snakes at their original point of capture within one day of surgery. During subsequent monitoring, no snakes exhibited overt negative responses (slow wound-healing, lethargy) to capture, surgery, or transmitter implantation.

We radio-tracked pythons between 24 November 2013 and 30 May 2016, locating the animals by using a Regal 2000 handheld receiver and 3-element Yagi antenna (Titley Scientific, Brendale, Australia). We located individual pythons at a wide range of times (both by day and at night), and frequently located snakes at multiple times on the same day to clarify short-term movement patterns. On average, we located each scrub python at least once every 1–2 weeks. The order in which we tracked animals both within and among days was changed frequently, to avoid temporal autocorrelation in the data^24^.

When a snake was located, we recorded the broad habitat type, the time of day, and the snake’s position to 5 m using a handheld GPS unit (Garmin GPSMAP^®^ 62 s). When possible, we also recorded the straight-line distance to the snake’s previous position using a flexible measuring tape, or by pacing the distance, to later check the accuracy of GPS estimates of distance. On many occasions snakes could not be visually sighted when located, due to being concealed within hollow logs, tree hollows, or in the tops of tall trees. When snakes could be sighted, we recorded their posture as resting, moving, or hunting following Natusch et al.^18^. Briefly, we considered pythons to be in ambush posture (hunting) if they were motionless with the head and neck in an “S” position (see also^25^); to be resting if they were tightly coiled; and to be moving if they were fully outstretched when encountered. To evaluate spatial ecology of snakes we separated telemetry data into two seasons: (1) the wet season (from 1 December to 30 April), and (2) the dry season (1 May to 30 November).

### Analyses of home range and use area

We initially tested several home range estimators on our data for scrub python spatial ecology, including minimum convex polygons (MCPs), kernel densities, and dynamic brownian bridge movements models (dBBMMs). All models have their limitations and assumptions, and their use depends on the tracking regime, species’ biology, and the questions being asked^26,27^. After testing several methods, we chose to calculate scrub python total home ranges using 100% MCPs. We considered MCPs to best represent total potential scrub python home range based on our experience tracking this species. Scrub pythons are capable of making large daily displacements and can utilize all of the contiguous habitat types in our remote study area. Therefore, although MCPs have been criticized because they sometimes include areas not actually used by the animal (and thus can overestimate range size e.g.^20^ and references therein), we considered those sites to be relevant even if use was not detected during the current study.

To complement estimates of total home range size, we also calculated 50% kernel densities to understand core use areas within this broader range. We chose the kernel smoothing factor (*h*) by first calculating 95% kernel densities and varying *h* until kernel area approximated the home range sizes calculated using the 100% MCP method. We then used the calculated *h*-values as the smoothing factor in our 50% kernel density estimates^28^. Although we report estimated range sizes for all snakes, we excluded individuals with < 20 fixes from our comparative analyses, as well as one individual where fixes occurred over a short period of time when the specimen was utilizing a spatially discrete resource hotspot (trees housing starling colonies^18^; Table 1). In addition, individuals were excluded from our analysis of core use areas if the animals restricted their movements to small areas for long periods of time, due to reproduction (clutch-brooding) or to use of resource hotspots (bird-colony trees). In these cases, overrepresentation of fixes at these sites had a disproportionate influence on the analysis (Table 1).

**Table 1:** Attributes (sexes, sizes) of radio-tracked scrub pythons (*Simalia amethistina*) in north Queensland, with data on the duration of radiotelemetric monitoring, number of times a snake was located (“fixes”) and home range sizes based on 100% minimum convex polygons (MCP) and core use areas based on 50% kernel density estimates.

Name	Sex	SVL (mm)	Mass (g)	Tracking start date	Days tracked	Fixes	Home range (100% MCP; km^2^)	Core use area (50% kernel density; km^2^)
Arwen^a^	F	2700	2892	24/11/13	50	14	0.125	–
Boromir	M	2400	2654	3/12/13	402	76	0.282	0.053
Galadriel	F	2930	4686	3/12/13	670	22	0.197	0.042
Saruman	M	3400	7385	3/12/13	351	20	0.485	–
Eowyn^a^	F	2410	1614	17/12/13	73	15	0.162	–
Gimli	M	2220	1495	21/12/13	769	90	0.595	0.09
Elbereth	F	2460	2507	21/12/13	1001	100	0.188	0.037
Pippin^a^	M	2120	1207	22/12/13	64	7	0.029	–
Nimrodel^a^	F	2370	1747	22/12/13	76	8	0.003	_–_
Goldberry	F	2800	2987	6/1/14	753	90	0.267	0.041
Gandalf	M	3320	6826	20/1/14	766	71	1.766	0.342
Arwen Dua	F	2720	2695	24/1/14	763	106	0.786	0.133
Legolas	M	3080	4565	24/1/14	586	119	0.298	0.056
Nimrodel Dua	F	2890	3915	22/4/14	703	73	0.384	0.065
Shelob	F	3130	5397	25/4/14	329	46	0.156	–
Loriel	F	3100	5009	24/5/14	88	21	0.149	0.03
Frodo	M	2760	4180	23/6/14	627	63	1.421	0.222
Sauron	M	3700	8686	23/6/14	436	51	0.438	0.052
Finis	M	2030	1030	3/12/14	287	42	0.54	–
Elfwink	F	3300	5000	15/12/14	451	38	0.416	0.086
Stumpy	F	2280	1838	15/12/14	632	72	0.233	–
Idril	F	2020	1107	17/12/14	568	51	0.139	0.022
Sam	F	2290	1518	5/1/15	87	41	0.017	–
Loriel Dua	F	2870	3077	8/1/15	405	46	0.286	0.065
Treebeard	F	2530	2029	12/1/15	90	40	0.37	–
Piggy^a^	F	2210	1179	19/1/15	319	60	0.035	–
Sam Dua^a^	F	2950	6000	15/7/15	177	24	NC	–

*SVL* snout-vent length.

^a^Denotes specimens for which MCP home range estimates should be viewed with caution due to the low number of fixes or altered movement behaviour (see “Methods”). These specimens were not included in our home range analyses.

We analyzed the data in R version 3.6.3 using the *adehabitatHR* package (R Core Team, Vienna, Austria, 2021), and used the ZoaTrack online program^29^ to visualize the data against satellite imagery to verify the accuracy of our estimates. We examined variation in home range and core use area sizes separately using analysis of covariance (ANCOVA) with snake sex as the factor, ln SVL as the covariate, and ln MCP and ln 50% kernel density estimates as the dependent variables. For snakes with sufficient fixes, we repeated the above analysis but included season (and its interaction) to examine seasonal difference in home range sizes. We performed these analyses in JMP Pro 14 (SAS Institute, Cary, NC).

### Analysis of habitat use

The only available habitat maps and shapefiles describing the vegetation of Cape York Peninsula are 25 years old and did not provide enough resolution to accurately examine habitat use by scrub pythons. Therefore, we manually recreated vegetation maps to improve habitat resolution based on the Queensland regional ecosystem guide and shapefiles provided by Neldner & Clarkson^21^. We overlaid MCPs from each snake onto these maps in QGIS (Quantum GIS Development Team, v. 3.2.1) and calculated the proportion of each habitat type within the polygon relative to the total home range area. We considered all habitats within those MCPs to be available for use. We then calculated the number of python fixes in each habitat type as a proportion of total fixes. To test if pythons used certain habitats disproportionately to their availability, we performed a chi-squared goodness of fit test with the proportion of available habitat within each snake’s MCP home range as the expected frequency and the proportion of fixes in each habitat as the observed frequency. We repeated this analysis to test for shifts in habitat use between wet and dry seasons by including the proportion of fixes in each habitat type in each season as the expected and observed frequencies.

### Analysis of activity and movement

We used nominal logistic regression to test the influence of season, time of day, sex, ln SVL, and their interactions on snake activity (resting, moving, hunting). We sequentially deleted non-significant interaction terms until we were left with main effects. To quantify movement patterns, we calculated mean daily movement distance for each telemetered snake as the total straight-line distance moved between successive fixes divided by the number of days between fixes. We analyzed movement distance using a linear mixed model (LMM) with season and sex as factors, ln SVL as a covariate, and ln movement distance as the dependent variable. Because we obtained multiple movement records per snake, we included snake ID as a random effect in the model to address pseudoreplication. We performed all analyses using JMP Pro 14 (SAS Institute, Cary, NC). Finally, because our calculation of mean daily movement distance relies on dividing total movement distance by the number of fixes obtained for each snake, and thus fails to include back-and-forth movements (net zero displacements), we also examined movement distances of a smaller sample of snakes (15) located on successive days and between night and day. We do not examine the resulting small number of records (58; i.e., 116 individual fixes) statistically, but we present them because they provide a more detailed understanding of individual movements.

### Ethical note

This research was carried out under Queensland Department of Environment and Heritage Protection permits (WISP12944313) and University of Sydney animal ethics committee guidelines (approval number: L04/3-2013/ 3/5969). All procedures involving animals were carried out in accordance with relevant guidelines and regulations (including ARRIVE guidelines).

## Results

We obtained 1406 fixes of 27 snakes tracked for a mean of 426 days (range 50–1001) over the course of this study (Table 1). Several snakes were tracked for only short durations due to expulsion of transmitters^30^ or natural mortality. Fixes were approximately evenly distributed between wet and dry seasons, but sample sizes varied among individual pythons. Of these fixes, 467 were made at night while 939 were made during the day.

### Home range and core use areas

**Figure 2:**
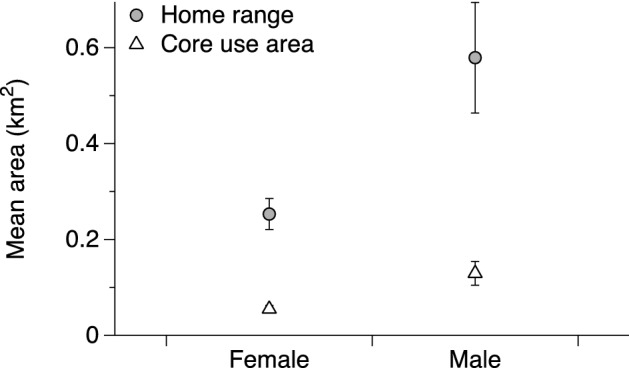
Mean home ranges and core use areas of male and female scrub pythons (*Simalia amethistina*) in Cape York. Home range size was calculated based on 100% minimum convex polygons and core use areas were calculated based on 50% kernel densities. See text for details.

Overall, home range sizes of scrub pythons averaged 0.38 ± 0.05 km^2^ (range: 0.17–1.77 km^2^; Table 1). Core use areas were fourfold smaller, averaging 0.09 ± 0.013 km^2^ (range = 0.1–0.34 km^2^; Table 1). Male scrub pythons had larger home ranges than did females (F_1,20_ = 6.58, P = 0.019), but used similar-sized core areas (F_1,15_ = 4.25, P = 0.06; Fig. 2). A snake’s body size did not significantly influence the size of its home range (F_1,20_ = 1.28, P = 0.273) or core use area (F_1,15_ = 0.46, P = 0.509).

For snakes with sufficient detections to examine seasonal effects, the mean home range was 143% (range: −6.7 to 533%) larger during the dry season than the wet season for males but only 6% (range: −40 to 118%) larger for females. However, due to small sample sizes this change was not statistically significant for either sex (males N = 5; F_1,10_ = 2.53, P = 0.15; females N = 6; F_1,11_ = 0.05, P = 0.82). There was considerable overlap in the home ranges and core use areas of multiple snakes in our study. At one site, eight telemetered pythons occupied part of another individual’s home range, along with numerous non-telemetered individuals located in this same area over the course of the study.

### Habitat use

Scrub pythons used five broad habitats, comprising six regional ecosystems (Table 2). The proportion of each habitat type within a snake’s home range varied among individuals, with several snakes using four different habitat types whereas others restricted their movements to rainforest throughout the study. Rainforest was the only habitat type used by all individuals, with snakes showing a clear preference for this habitat type. Our chi-squared analyses revealed that 41% (7/17) of snakes used closed forest habitat at a higher rate than expected from its availability, whereas the remaining animals either showed no significant preference (41%) or preferred woodland, swamp, or sand plains (18%; Table 2). Several (6/11) individuals with sufficient fixes in each season showed significant seasonal shifts in habitat use. Four snakes used rainforest in the wet season but moved to open habitats (woodland, swamp and sand plains) in the dry season, whereas the other two individuals showed the opposite pattern (Table 2).

**Table 2:** Habitat associations of scrub pythons (*Simalia amethistina*) in Cape York showing the relative proportion of each habitat type in the home range of telemetered pythons and the proportion of total fixes that were made in those habitat types.

Snake ID	Sex	Home range (km^2^)	Rainforest (RF)		Woodland (WL)		Swamp (SW)		Sand plains (SP)		Heathland (HL)		Habitat selection	P-value from χ^2^ test	Habitat shift	P-value from χ^2^ test
			Area	% Fixes	Area	% Fixes	Area	% Fixes	Area	% Fixes	Area	% Fixes				
Boromir	M	0.282	35.5	38.1	53.9	48.7	10.6	13.2	0	0	0	0	None	0.52	RF wet → SW dry	< 0.0001
Galadriel	F	0.197	76.1	76.2	23.9	23.8	0	0	0	0	0	0	None	0.99	None	0.63
Saruman	M	0.485	57.7	85.7	42.3	14.3	0	0	0	0	0	0	RF	< 0.0001	–	–
Gimli	M	0.595	18.5	64.1	76.5	34.6	5.0	1.3	0	0	0	0	RF	< 0.0001	RF wet → WL dry	< 0.0001
Elbereth	F	0.188	99.5	98.0	0.5	2.0	0	0	0	0	0	0	None	0.92	None	0.99
Goldberry	F	0.267	37.5	57.8	62.5	42.2	0	0	0	0	0	0	RF	< 0.0001	WL wet → RF dry	< 0.0001
Gandalf	M	1.766	83.0	95.3	17.0	4.7	0	0	0	0	0	0	RF	0.002	None	0.09
Arwen Dua	F	0.786	10.2	37.7	66.9	42.5	11.4	19.8	11.4	0	0	0	RF, SW	< 0.0001	None	0.077
Legolas	M	0.298	40.3	29.1	59.7	70.9	0	0	0	0	0	0	WL	0.029	None	0.25
Nimrodel 2	F	0.384	97.4	93.2	2.6	6.8	0	0	0	0	0	0	None	0.0187	–	–
Shelob	F	0.156	76.9	73.7	23.1	26.3	0	0	0	0	0	0	None	0.51	–	–
Loriel	F	0.149	68.4	65.0	32.9	35.0	0	0	0	0	0	0	None	0.65	–	–
Frodo	M	1.421	63.4	20.6	26.7	57.1	4.9	7.9	4.9	14.3	0	0	WL, SP	< 0.0001	RF wet → WL,SP dry	< 0.0001
Sauron	M	0.438	1	1	0	0	0	0	0	0	0	0	100% RF	–	–	–
Finis	M	0.54	72.7	89.6	18.2	8.3	0	0	0	0	9.1	2.1	RF	0.0007	–	–
Elfwink	F	0.416	1	1	0	0	0	0	0	0	0	0	100% RF	–	–	–
Stumpy	F	0.233	77.6	82.2	13.4	13.7	0	0	0	0	9.0	4.1	None	0.23	–	–
Idril	F	0.139	40.0	51.0	64.0	49.0	0	0	0	0	0	0	RF	0.0025	RF wet → WL dry	< 0.0001
Sam	F	0.017	1	1	0	0	0	0	0	0	0	0	100% RF	–	–	–
Loriel 2	F	0.286	62.9	59.1	35.4	34.1	1.7	6.8	0	0	0	0	SW	0.0003	WL wet → RF dry	< 0.0001
Treebeard	F	0.37	1	1	0	0	0	0	0	0	0	0	100% RF	–	–	–
Piggy	F	0.035	1	1	0	0	0	0	0	0	0	0	100% RF	–	–	–

Habitats selected more frequently than expected from their overall availability are shown together with the P-values for χ^2^ significance tests. If no habitat preference was observed for an individual python it is recorded as ‘none’. If the analysis could not be completed because only one habitat type was available in the home range it is recorded as ‘100% rainforest’. Seasonal shifts in habitat use and the direction of the shift are shown, along with the P-values for the χ^2^ significance test. For example, ‘RF wet → SW dry’ records a habitat shift from rainforest in the wet season to swamp in the dry season. Regional Ecosystems for each habitat type are provided below the table. See “Methods” for details of the calculation of these results.

RF: Regional ecosystem 3.5.3 (Semi-deciduous notophyll vine forest of the Carnegie tableland) and 3.2.1 (Evergreen notophyll vine forest in coastal dunefield systems).

WL: Regional ecosystem 3.5.5(a) (*Corymbia novoguinensis* + /− *C. tessellaris* woodland on sand plains on northern Cape York Peninsula).

SW: Regional ecosystem 3.3.5 (*Melaleuca* spp. woodland on swamps on floodplains).

SP: Regional ecosystem 3.5.14 (*Melaleuca viridiflora* + /− *Acacia* spp. + /− *Asteromyrtus symphyocarpa* low woodland on scattered coastal sand plains).

HL: Regional ecosystem 3.2.33 (*Gahnia sieberiana* open to closed heath in drainage swamps in east coast dunefields).

### Activity patterns

**Figure 3:**
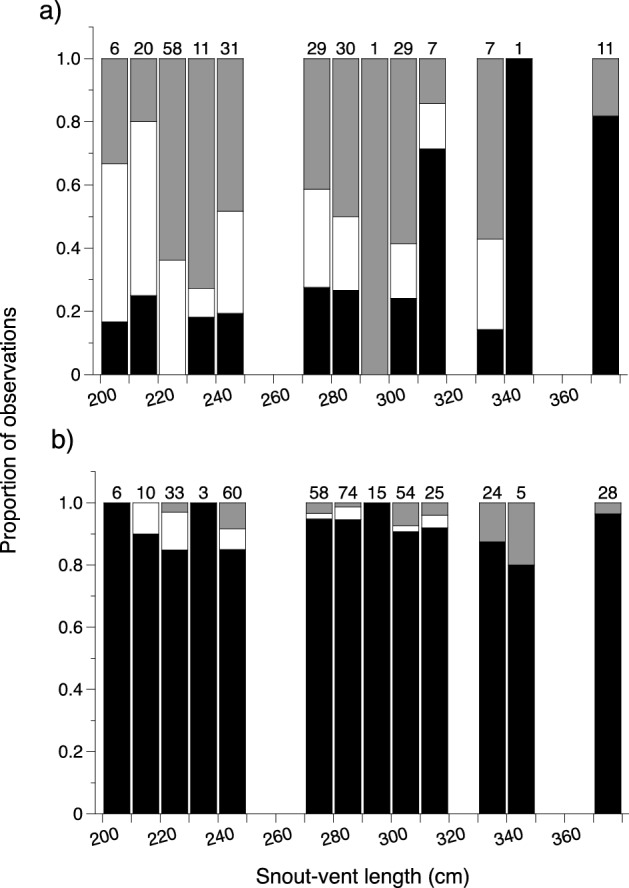
Proportion of observations of scrub pythons resting (black columns), moving (white columns), or hunting (grey columns) at (**a**) night and (**b**) during the day based on the snout-vent length of the snake. The number of observations made in each size class appear above the columns.

After deletion of non-significant three- and two-way interactions, our nominal logistic regression on main effects revealed that python activity was influenced by the time of day that snakes were located (χ^2^ = 252, df = 2, P < 0.0001), by their body size (χ^2^ = 17.5, df = 2, P = 0.0002), and by the season in which they were located (χ^2^ = 6.1, df = 2, P = 0.048). Scrub pythons were primarily nocturnal at our study site. When we located telemetered snakes by day, they were typically resting whereas at night they were often moving or hunting (Fig. 3). Overall, larger snakes spent less time hunting than small snakes, but hunted more often during the day (Fig. 3). Pythons made most movements around dusk (1800 to 2000 h) and retreated to their diurnal resting sites around dawn (0600 to 0800 h). The snakes were most often found hunting during the wet season and resting during the dry season, with no significant influence of sex (χ^2^ = 0.05, df = 2, P = 0.97) on activity patterns.

### Movements

**Figure 4:**
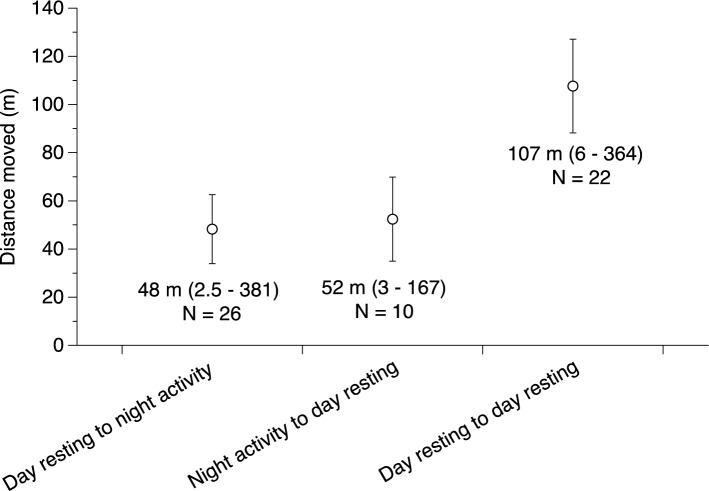
Mean movement distances of telemetered scrub pythons (*Simalia amethistina*) from daytime resting sites to nocturnal hunting sites and back again. Means and movement ranges are provided below each data point.

Our radio-tracked scrub pythons moved an average of 9.8 ± 1.1 m/day (range: 1.7–36 m/day). After deletion of non-significant interactions, our linear mixed model revealed no significant difference in daily displacements between sexes (F_1,38_ = 0.19, P = 0.67), seasons (F_1,38_ = 2.83, P = 0.11) or snakes of different body sizes (F_1,38_ = 0.29, P = 0.60). However, the overall mean displacement of less than 10 m per day is somewhat misleading, in that most snakes moved back-and-forth between refuges and ambush sites. Examination of night-day and day-day movements over known time periods (and thus, incorporating back-and-forth movements) saw an increase in mean daily movement distance to 107 m per day and approximately half that for movement between diurnal and nocturnal resting sites (Fig. 4). The longest single daily movement recorded was 364 m (Fig. 4).

Movements from diurnal resting sites to nocturnal ambush sites were typically small, and sometimes only a few metres (Fig. 4). Rather than moving from their daytime resting position, snakes often simply uncoiled the anterior portion of the body and set up ambush at their resting location. On other occasions, snakes resting in tree hollows during the day emerged to hunt in the same tree, either in the branches or near the hollow itself. Movement distances from nocturnal hunting sites back to resting positions were equally small (Fig. 4). By far the most common pattern was for snakes not to move at all from one day to the next.

## Discussion

Our results are consistent with previous work on this species^8,18^, and with telemetry-based studies on other large sit-and-wait predatory snakes (e.g., viperids^31–34^). In strong contrast to snakes from the cool-temperate zone, which often migrate long distances from winter hibernacula to summer feeding ranges^35,36^, warm conditions year-round in the Lockerbie Scrub mean that seasonal shifts by pythons were minor, often involving movements between adjacent habitat types. In tropical systems where pronounced wet-dry seasonality generates flooding that redistributes prey across the landscape, predatory snakes also may move considerable distances^37^. In the Lockerbie Scrub, in contrast, wet-season rains do not cause major flooding over most areas; and hence, the seasonally-shifting variables that affect prey availability for scrub pythons likely involve issues such as vegetation density (and hence, the effectiveness of camouflage and ambush predation) and seasonal reproduction of mammalian and avian prey (generating offspring accessible to pythons^18^). As a result, heterogeneity in prey availability likely varies over a small spatial scale in this system, favouring only minor movements between adjacent habitat types rather than broadscale migration across the landscape. Regular movements over long distances may be more important for active-searching predators; once they have checked all available prey refuges in an area, the optimal tactic may be to move further afield^23^. In contrast, a generalist ambush predator such as a scrub python is unlikely to consume enough prey items to substantially deplete overall feeding opportunities in a local area.

Requirements for reproduction can also drive changes in habitat use; for example, gravid females of many viviparous snake species in cool climates accelerate developmental rates of their embryos by congregating in sun-exposed (and thus warm) microhabitats^38–40^. The movement of scrub pythons from rainforest to woodlands in our study, during the dry season when reproduction occurs, may also be for this reason. Nevertheless, we recorded mating in both open woodland and rainforest habitats during the course of our study. Three radio-tracked female pythons built nests and attended their broods until hatching, all within rainforest habitat. However, the juxtaposition of woodland and rainforest within our study area, with most pythons utilizing interface habitats (Fig. 1), meant that a snake could select a rainforest nesting site without moving far from any location within its home range. In essence, the Lockerbie Scrub contains a mosaic of habitat types, which may differ slightly in their suitability for feeding and breeding, but variation occurs over too small a spatial scale to necessitate long-distance movements on either a diel or seasonal basis.

The scarcity of long-distance displacements in our radio-tracked snakes hints that movements may be costly and/or dangerous. Intuition suggests that such large animals would be invulnerable to predation, but there are several reports of relatively large Australian pythons being killed and consumed by canids such as dingos^41,42^. Additionally, moving is incompatible with ambush predation. Not only does movement render a snake conspicuous to potential prey, but it also may leave scent trails that are detectable by prey animals^43^; and ambush predators rely upon being undetected^44^.

The primary effects of season were to increase home range sizes of our snakes (although not statistically significant), to increase hunting effort (proportion of time spent in ambush-foraging pose), and to alter the habitat of some (but not all) snakes. The heightened effort at hunting in the wet season suggests that the expansion in home range sizes of male snakes in the dry season reflects mate-searching, as recorded in many other snake species^23,45^. Diel cycles in behaviour were pronounced, with snakes (especially smaller individuals) usually inactive by day, often hidden within refuges (Fig. 3). Most of the mammals consumed by scrub pythons are primarily nocturnal^18^, such that night offers the best opportunity for an ambush predator to seize a moving prey item. Larger species such as agile wallabies (*Macropus agilis*) sometimes are active by day as well^46^, such that a large python (the only size class capable of consuming such a large item) may benefit by extending its foraging effort into daylight hours (Fig. 3). Interestingly, the trend for increased diurnal hunting by larger snakes is opposite to that seen in a closely-related python species from cooler climates: around Sydney, juvenile diamond pythons (*Morelia spilota*) ambush by day (unlike conspecific adults), apparently because suitably small prey (lizards) are diurnally active, and because nights are too cool for a small python to retard heat loss for long enough to remain an effective predator^47^. Those constraints are not applicable to scrub pythons: even hatchlings are large enough to feed on endothermic prey, and the tropical climate of the Lockerbie Scrub allows snakes to retain high body temperatures throughout the night.

Perhaps the most important facet of our results is the flexibility of python ecology, as exemplified by strong variation among individual snakes. Thus, for example, some snakes moved into the rainforest in the dry season, whereas other individuals went the other way. As reported in other studies of snakes (e.g.,^48^), minor differences in habitat attributes and/or behavioural traits of individuals generate substantial inter-individual variation in spatial ecology. Those differences are amplified by flexible responses to local conditions. In the case of scrub pythons, such flexibility is exemplified by facultative use of resource hotspots, whereby snakes remain within small areas and shift to active searching rather than ambush foraging under trees containing bird colonies^18^. The same flexibility is evident in the success of this species in highly disturbed suburban and urban environments, where the snakes take refuge within buildings and feed on commensal mammals and birds^49^.

Our results are encouraging for the conservation of these giant pythons. Even large individuals required only small areas, and tolerated high levels of overlap with conspecifics. Flexible use of habitats—as exemplified by urban pythons as well as by free-ranging snakes utilizing both dense and open forests—renders this species resilient to habitat change. As long as suitable endothermic prey are common, a scrub python can thrive even in a small habitat patch, because it can adjust its behaviour (habitat selection, activity pattern, diet choice, foraging tactics) to exploit the opportunities available (e.g.,^18^). This is exemplified by the persistence of scrub pythons on small sand islands in Cape York (Milman Islet; 0.22 km^2^—the size of a single python home range^49^). Such flexibility is common in many snakes, but is especially evident in giant tropical species because the large range of body sizes within a single species creates wide variation in traits such as dietary composition and reproductive output (e.g.,^6^), and reduced thermal constraints in tropical habitats provide a greater opportunity for flexible adjustment of behaviour to resources.

Although the scientific literature on snake ecology remains strongly focused on small-to-medium-sized species in cool-temperate habitats, recent years have seen a welcome expansion of study systems in terms of body sizes, phylogenetic affiliations, and geographic locations. Thus, for example, recent work has documented the spatial ecology of large venomous snakes both on land (*Ophiophagus hannah*^50,51^) and underwater (*Hydrophis curtus*^52^) as well as pythons and boids both in their native ranges (*Eunectes marinus*^53^; *Malayopython reticulatus*^54^; *Python natalensis*^55^) and in areas that they have invaded (*Python bivittatus*^56^). This renaissance suggests that we may soon be able to interpret the ecology and conservation needs of giant tropical snakes from actual field data on those animals, rather than by sometimes-unreliable extrapolation from studies based on smaller species from different climatic zones.

## Data Availability

Data will be deposited in the Dryad repository upon manuscript acceptance.
